# Supplementary material for: Shallow water heterobranch sea slugs (Gastropoda: Heterobranchia) from the Región de Atacama, northern Chile
Source: PeerJ. 2016 May 2;4:e1963. doi: 10.7717/peerj.1963 (PMC4860336; doi:10.7717/peerj.1963)
Supplement: Supplemental Information 1 — Resumen en Español. [file peerj-04-1963-s001.docx]

**resumen**

La costa del norte de Chile ha sido escasamente estudiada con respecto a su fauna de invertebrados, con solo unos pocos estudios revisando la distribución de moluscos locales. Este trabajo presenta un estudio de campo de las babosas marinas heterobranquias de aguas someras presentes alrededor del puerto de Caldera (27º S), en la Región de Atacama, norte de Chile. Ocho especies de babosas marinas fueron encontradas en este estudio: *Aplysiopsis* cf. *brattstroemi* (Marcus, 1959), *Baptodoris* *peruviana* (d’Orbigny, 1837), *Diaulula* *variolata* (d’Orbigny, 1837), *Doris* *fontainii* d’Orbigny, 1837, *Onchidella* *marginata* (Couthouy in Gould, 1852), *Phidiana* *lottini* (Lesson, 1831), *Tyrinna* *delicata* (Abraham, 1877) y la nueva especie *Berthella* *schroedli* sp. nov., descrita en el presente trabajo. Todas las especies encontradas en el área son endémicas para Sudamérica, teniendo distribuciones en el Pacífico Suroeste y en el Sur Atlántico, desde Ancash, Perú hasta Península Valdés, Argentina, y dos especies de ellas representan especies endémicas para las costas Chilenas (*Aplysiopsis* cf. *brattstroemi* y *Berthella* *schroedli*). El registro de una especie previamente no descrita enfatiza la necesidad de futuros estudios de campo, particularmente en el submareal y en aguas más profundas, esenciales para mejorar el conocimiento de esta fauna en Atacama.
